# Development of a novel gene expression panel for the characterization of MSCs for increased biological safety

**DOI:** 10.1007/s13353-024-00917-5

**Published:** 2024-12-02

**Authors:** Anna M. Różycka-Baczyńska, Igor M. Stepaniec, Marta Warzycha, Izabela Zdolińska-Malinowska, Tomasz Oldak, Natalia Rozwadowska, Tomasz J. Kolanowski

**Affiliations:** 1 Research and Development Department, Polski Bank Komórek Macierzystych S.A. (FamiCord Group), Warsaw, Poland; 2 Institute of Human Genetics, Polish Academy of Sciences, Poznan, Poland

**Keywords:** Cell therapy, Mesenchymal stromal cells, MSCs, Oncogenic potential, Cancer risk assessment

## Abstract

Mesenchymal stromal cells (MSCs) have a wide range of therapeutic applications due to their multipotency, immunomodulatory, and anti-inflammatory properties. Their ability to migrate and recolonize damaged tissues is also remarkable. However, the controversial occurrence of spontaneous tumorigenesis or malignant transformation of MSCs raises concerns about proposed cell-based therapies for patients that researchers must address. There are several in vitro and in vivo strategies for MSC safety approval, but there is still no described coherent scheme that allows the assessment of MSC oncogenic potential in a simple, robust, and reproducible manner. Here, we have developed a diagnostic panel of molecular markers that allows for the accurate verification of the quality and safety of MSCs. Moreover, presented in this article diagnostic panel that can define the origin and tumorigenicity of MSCs can be easily introduced into the routine quality control processes of MSC-based product manufacturing which will improve further clinical applications of MSCs.

**Graphical Abstract:**

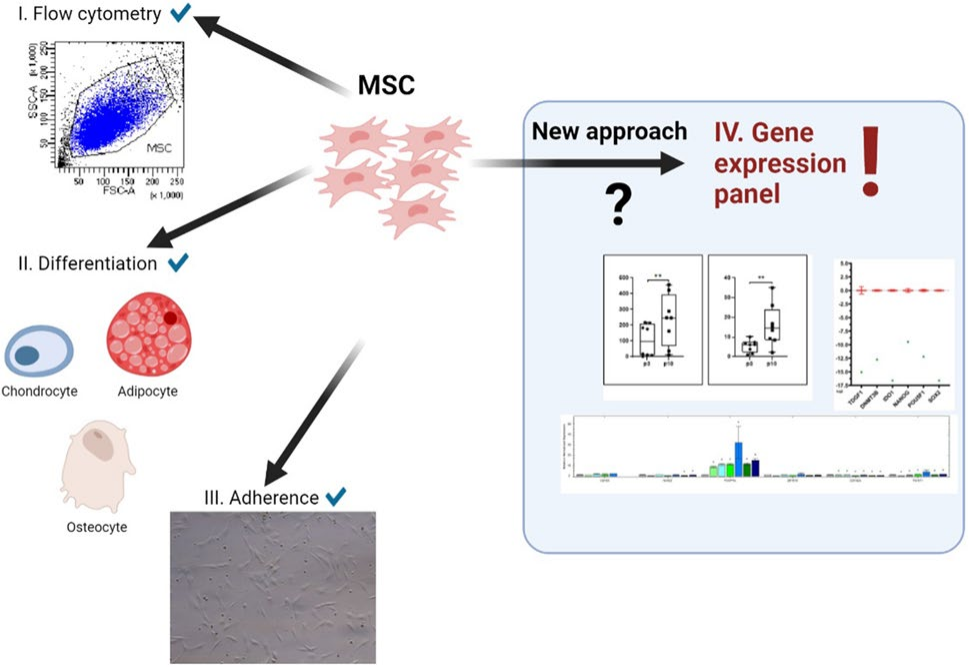

**Supplementary Information:**

The online version contains supplementary material available at 10.1007/s13353-024-00917-5.

## Introduction

Mesenchymal stromal cells (MSCs) were first characterized by Friedenstein et al. (1976). Subsequent research proved their nonhemopoietic origin and showed that they are undifferentiated and multipotential adult progenitor cells (Bianco 2014). Recent studies (Pittenger et al. 2019) clarify that MSCs are an actual heterogeneous population composed of multipotent stem cells, already differentiated cells, and their initial progenitors. Therefore, only a fraction of MSCs show an ability for self-renewal and differentiation into three mesodermal cell lineages: osteoblasts, chondroblasts, and adipocytes.

In contrast to stem cells such as embryonic stem cells (ESCs) or induced pluripotent stem cells (iPSCs), MSCs have limited proliferation potential with a capacity of approximately 40–60 population doublings (Dominici et al. 2006). Under appropriate conditioning or induction by enriched media, MSCs can differentiate into other types of cells: progenitor muscle cells (Nöth et al. 2010), hepatocytes (Seo et al. 2005), and neuroblasts (Krampera et al. 2007).

MSCs are observed in two morphological forms when cultured *in vitro*. The most typical form is a spindle-like fibroblast form occurring during aging or stress. Despite morphological differences, both forms have similar properties, i.e., the ability to adhere to plastic surfaces; differentiate into three cell types, namely, chondroblasts, osteoblasts, and adipocytes; and express surface markers, such as CD105, CD73, and CD90, without expressing hematopoietic markers, such as CD45, CD34, CD14 or CD11b, HLA-DR, and CD79-α or CD19. These criteria defining MSCs were described more than a decade ago by The International Society for Cellular Therapy (ISCT) (Dominici et al. 2006). Due to their proliferative abilities and secretion of anti-inflammatory molecules, MSCs can be considered powerful tools in cell-based therapy development. Accordingly, the application of MSCs is currently being investigated in hundreds of registered clinical trials (Levy et al. 2020). In recent years, evidence has shown that the previously described minimum criteria are not sufficient, as MSCs show interpopulation heterogeneity due to (a) donor characteristics, (b) tissue origin, and (c) isolation and *in vitro* culture methods (McLeod and Mauck 2017). To make therapies safer and more homogenous, a new methodological algorithm called DOSES, where D means donor (i.e., autologous, allogeneic, or xenogeneic), O—origin tissue, S—separation method, E— exhibited characteristics (associated with cell behavior), and S—site of delivery, has been proposed (Galderisi et al. 2022).

MSCs have been isolated from a plethora of tissues from both adults and fetal individuals (Abdulrazzak et al. 2010). In fetuses, MSCs colonize umbilical cord blood, Wharton’s jelly, and placenta (Kobolak et al. 2016). In adults, MSC populations can be identified in most organs, including the lungs, bones, fat tissue, bone marrow, and skeletal muscles (Barkholt et al. 2013). Despite the wide spectrum of niches in which MSCs can reside, cells derived from bone marrow, adipose tissue, and especially fetal tissues are the most promising for use in clinical therapies. Transplantation of advanced therapy medical products (ATMPs) may cause an increased risk of oncogenic transformation not only by the cells themselves but also as a result of their anti-inflammatory properties and cross-contamination of MSC preparations (Barkholt et al. 2013, Lee and Hong 2017). As MSCs age in long-term *in vitro* cultures, accumulation of DNA aberrations may occur, increasing the probability of oncogenic transformation (Barkholt et al. 2013, Sato et al. 2019). A few reports suggested that MSCs can spontaneously transform into tumor cells (Rubio et al. 2005); however, all of those findings were verified to be a result of cross-contamination with transformed cells (Torsvik et al. 2010). Taking this into consideration, the importance of recommending that cell culture is performed under good manufacturing practice conditions to ensure proper separation, cell identity, and control of starting and raw materials should be emphasized.

Specific standardization was introduced when MSCs were qualified as ATMPs. To date, there is no globally accepted strategy regarding the evaluation of potential MSC tumorigenicity and further steps are needed for proper risk assessment. A deeper understanding of the factors contributing to the tumorigenicity of MSCs is essential to their safe clinical application. It needs to be highlighted that interactions with the tumor microenvironment may promote tumorigenic behavior, but the exact mechanisms remain to be clearly defined (Papait et al. 2020).

To overcome these inadequacies, we designed a genetic panel of molecular markers that allows verification of the quality of MSCs. To prove that MSCs do not express pluripotency genes or uncontrolled self-renewal-associated genes, appropriate markers were included. The implemented protocol can be used to distinguish the gene expression profile of MSCs from human iPSCs and cancer cells of different origins. The proposed panels have the potential to extend the ISCT basic criteria for the identification of MSCs. As MSC-based treatments continue to expand in the field of regenerative medicine, ensuring their safety is necessary and this study represents an important step toward achieving that goal.

## Materials and methods

### Cell isolation and in vitro culture

Umbilical cords (UCs) were collected by trained midwives after cesarean sections and natural deliveries after informed consent was received. Tissues were obtained according to European Medicine Agency (European Medicines Agency 2008) guidelines regarding human cell-based medicinal products. UCs were processed following an internal standard operating procedure (SOP) approved by the Polish Main Pharmaceutical Inspectorate. MSCs were isolated mechanically as previously described (Semenova et al. 2017). Tissue fragments were kept under standard conditions in xenofree, GMP-approved NutriStem® MSC XF medium (Biological Industries) supplemented with 1% antibiotic-antimycotic solution (Thermo Fisher Scientific), 37 °C, 90% humidity, and 5% CO2. Primary cultures of MSCs obtained from umbilical cords of 10 different donors (*n* = 10) were cultured *in vitro* under analogous conditions. MSCs were passaged when they reached approximately 80% confluence. Cells were detached using TrypLE™ Express Enzyme (Gibco), and their viability and number were analyzed with an ADAM MC 2.0 cell counter. Cells with at least 95% viability from p3 and p10 were used in experiments.

Commercially available human cancer cell lines originating from all three germ layers endoderm: NCI-H727 (no. CRL-5815, Lot: 18H025, ATCC), K1 (no. 92030501, Lot: 14F004, Merck), and A-549 (no. 86012804, Merck); mesoderm: MG-63 (no. 86051601, Lot: 14K002, Merck) and HT-1080 (no. CCL-121, Lot: 63835574, ATCC); and ectoderm: A-375 (no. 88113005, Merck), A-431 (no. 85090402, Lot: 19A004, Merck), MCF-7 (no. HTB-22, ATCC), and ZR-75-30 (no. CRL-1504, Lot: 13D024, ATCC) were purchased and cultured according to supplier recommendations. MSCs and cancer cells were washed twice with Dulbecco’s phosphate-buffered saline (DPBS, Gibco), pelleted, shock frozen in liquid nitrogen, and stored at −80 °C for RNA isolation.

### Cell differentiation

To assess the capability of isolated cells to differentiate, appropriate differentiation protocols were used. MSCs (p2) were seeded at a density of 5000/cm^2^ into 24-well plates. To induce differentiation, cells were cultured in StemPro™ adipogenesis, chondrogenesis, and osteogenesis differentiation media. MSCs were cultured under standard conditions (37 °C, 90% humidity, and 5% CO₂); differentiation medium was changed every 2–3 days for 3 weeks followed by cell fixation in 4% paraformaldehyde solution, washing in DPBS and a staining procedure. Characteristics of cell type features were visualized using Oil Red O for lipid droplets in adipocytes, Alizarin Red for early calcium deposits in osteoblasts, and Alcian Blue for glycosaminoglycans and mucopolysaccharides in chondroblasts. Specimens were observed under a Leica DMIL light microscope.

### Flow cytometry

MSCs were incubated for 30 min in BD Horizon Brilliant™ Stain Buffer with BV421-conjugated antibodies against the human antigens CD73 and CD90 (BD Horizon™, BD Biosciences), Alexa Fluor® 647-conjugated antibodies against the human antigen CD105 (BD Pharmingen, BD Biosciences), and FITC-conjugated antibodies against the human antigens CD34 and CD45 (BD Bioscience). Cells were tested with an isotype-matched control antibody (BD Bioscience). Samples were run on a FACS Celesta flow cytometer (BD Bioscience) and analyzed with BD FACSDiva™ Software.

### RNA isolation: integrity and quality check

Total RNA from MSCs and cancer cell lines was isolated using the RNeasy Mini Kit (Qiagen) according to the manufacturer’s protocol. The purity of the samples was analyzed spectrophotometrically by measuring absorption at OD 260/280 nm and OD 260/230 nm on a BioDrop µLITE machine (BioDrop). To analyze RNA integrity, 500 ng of RNA was stained with Midori Green (ABO) and run for 1 h on a 2% agarose gel at 4 °C (75 V). The gels were UV visualized in a ChemiDoc XRS+ Imaging System (Bio-Rad). Two sharp bands (at a ratio of approximately 2:1, indicating 28S rRNA and 18S rRNA) and no smears indicated that the RNA samples were intact. iPSC RNA was purchased from Science Cell and treated under manufacturer recommendations.

### Reverse transcription

RNA samples of good quality and integrity were used to synthesize cDNA. To perform RT-PCR, the RevertAid First Strand cDNA Synthesis Kit (Thermo Fisher Scientific) with random hexamer primers was used. The reaction using 500 ng of RNA for each sample was carried out using a C1000 thermal cycler (Bio-Rad) following the manufacturer’s instructions. The obtained cDNA samples were stored at –80 °C.

### Primer design for custom-designed assay

Specific primers (PROM1, CDKN2A, FUT3, TDGF1, HER2, SOX9, B4GALNT1, TWIST1, GAPDH, and HPRT1 genes) for RT-PCR were designed according to standard procedures using NCBI primer BLAST and NCBI Gene base software. Primer thermodynamic analysis was performed with NetPrimer software using the nearest neighborhood algorithm. The list of primers is presented in Supplementary Table 1. The quality comparison and confirmation of primer utility were performed during electrophoresis separation on a high-resolution agarose gel. In each case, the experiment resulted in a single-band product of the expected length.

### Real-time PCR

RT-PCRs were carried out using a CFX-96 thermal cycler (Bio-Rad). Primers were used at a 4 mM concentration in nuclease-free water. Other reagents, such as POWERUP SYBR® master mix, nuclease-free water, and cDNA, were added. The gene expression study included cell line evaluation by custom-designed PrimePCR™ Assay plates (Bio-Rad) according to the instructions manual and regular qRT-PCR for genes presented in Supplementary Table 1. Genes included in the PrimePCR™ Assay are listed in Supplementary Table 1 and were based on gene panels contained within the hPSC Score-card™. Reactions were performed with two or three technical replicates (for PrimePCR™ Assay and primers, respectively). The following thermocycling conditions were used: 50 °C for 2 min and 95 °C for 2 min followed by 40 cycles of 95 °C for 15 s and 60 °C for 1 min. Data were analyzed with Maestro 1.1 software (Bio-Rad).

To calculate the relative gene expression, we used the 2-ΔΔCT method (Livak and Schmittgen 2001). Gene expression was normalized against the reference genes GAPDH and HPRT1 or GAPDH and ACTβ for PrimePCR™ Assay plates. Due to the number of genes analyzed, interplate controls were added based on one of the MSC samples and housekeeping genes such as GAPDH, ACTβ, and HPRT 1. Gene expression data for tumor cell lines for the mesoderm panel are averaged results from 3 tumor cell lines derived from mesoderm, two technical replicates each. For the endoderm gene expression panel, averaged results from 3 tumor cell lines derived from endoderm were used, two technical replicates each. For ectoderm gene expression panel, 3 tumor cell lines derived from ectoderm were used, two or three replicates each.

### Statistical analysis

Gene expression values (2-ΔΔCT) relative to biological groups (average expression of genes from MSC lines, iPSC lines, and cancer cell lines) are presented in log2 to obtain a symmetrical distribution. Statistical significance was calculated by the nonparametric Mann-Whitney test, and *p* values lower than 0.05 were considered significant. Gene expression graphs were generated, and statistical analysis was performed with GraphPad Prism 8.0.

## Results

### Multilineage differentiation

**Fig. 1:**
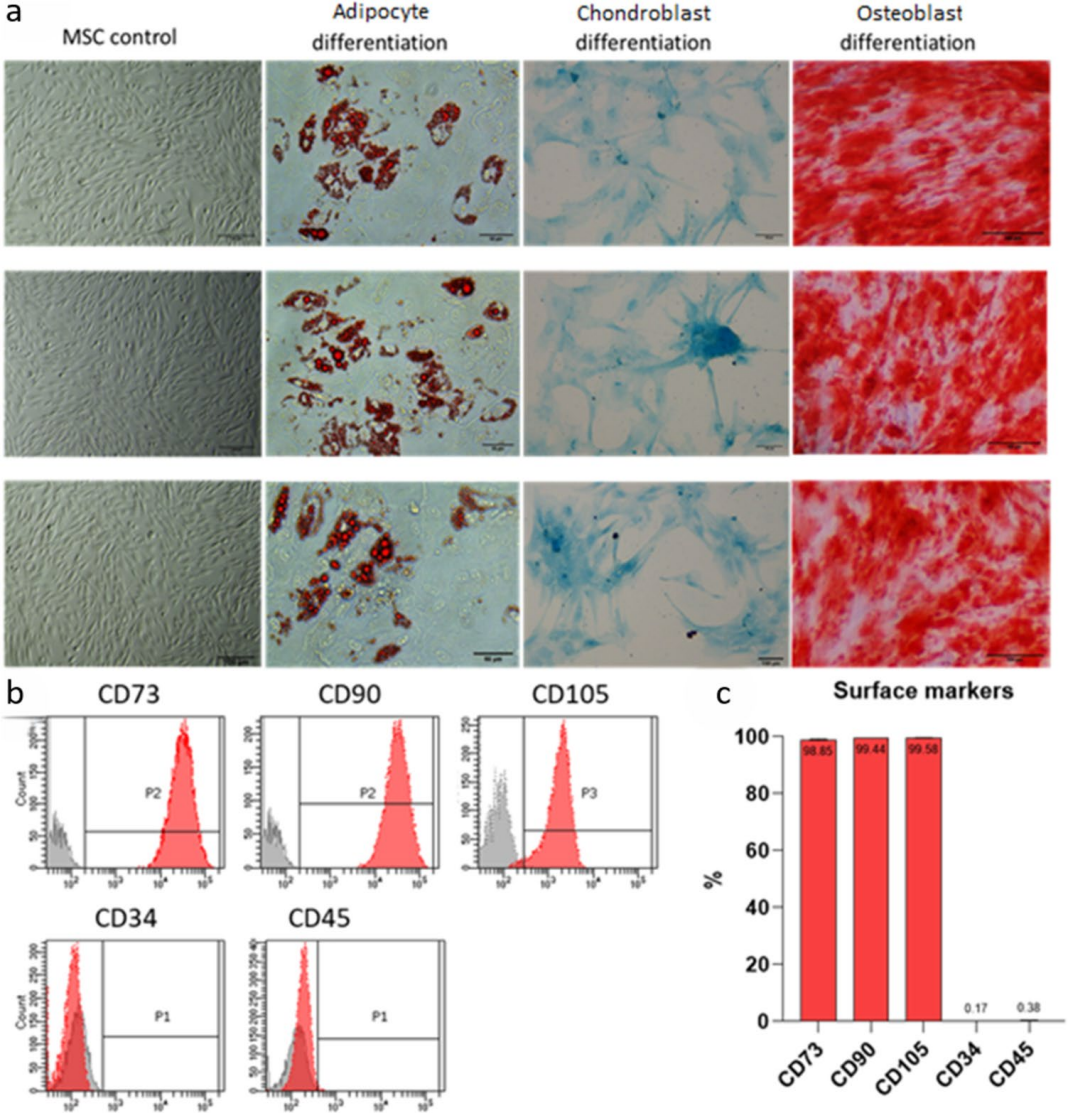
Multilineage differentiation and immunophenotype characterization at passage 3 (p3) (*n* = 10). **a** Representative images from three different MSC donors in conventional culture: unstained control (left-most panel), Oil red O showing differentiation into adipocytes (left panel), alcian blue showing differentiation into chondroblasts (right panel), and Alizarin red showing differentiation into osteoblasts (right-most panel). Size bar 50 µm for images showing differentiation into adipocytes and 100 µm for the other images. **b** Representative flow cytometry analysis of the surface markers CD45, CD34, CD90, CD73, and CD105 for MSCs used in further experiments (red peaks). The isotype controls are represented by the gray peaks. **c** The percentage of surface marker expression from different MSCs

MSCs were obtained from 10 donors of Wharton’s jelly (WJ-MSCs) from the umbilical cord. The isolated cells were assessed for capability to differentiate into three types of cells: osteoblasts, adipocytes, and chondroblasts. After 3 weeks of *in vitro* culture in differentiation media, cells from all donors changed their morphology, and lipids, calcium deposits, and glycosaminoglycans appeared and were visualized with proper cytological staining (Fig. 1a).

### Immunophenotype characterization

The phenotype analysis of cultured MSCs at passage 3 (p3) (*n* = 10) showed that over 98% of cells demonstrated surface expression of CD73, CD90, and CD105. The analysis revealed negligible expression of CD34 and CD45, which was detected in less than 1% of events (Fig. 1b, c). These cell populations were used for further experiments, and none of the donors was rejected due to nonoptimal expression levels of surface markers.

### Gene expression assessment

We designed an RT-PCR-based assay to provide basic molecular characteristics of MSCs and distinguish MSC expression profiles from both iPSCs and cancer cell lines. Our approach included gene markers specific to pluripotent stem cells (9 genes), mesendoderm (5 genes), other (6), and the three embryonic germ layers, endoderm (EN, 25 genes), mesoderm (ME, 22 genes), and ectoderm (EC, 22 genes). Two housekeeping genes were used as references and for interplate data normalization.

**Fig. 2:**
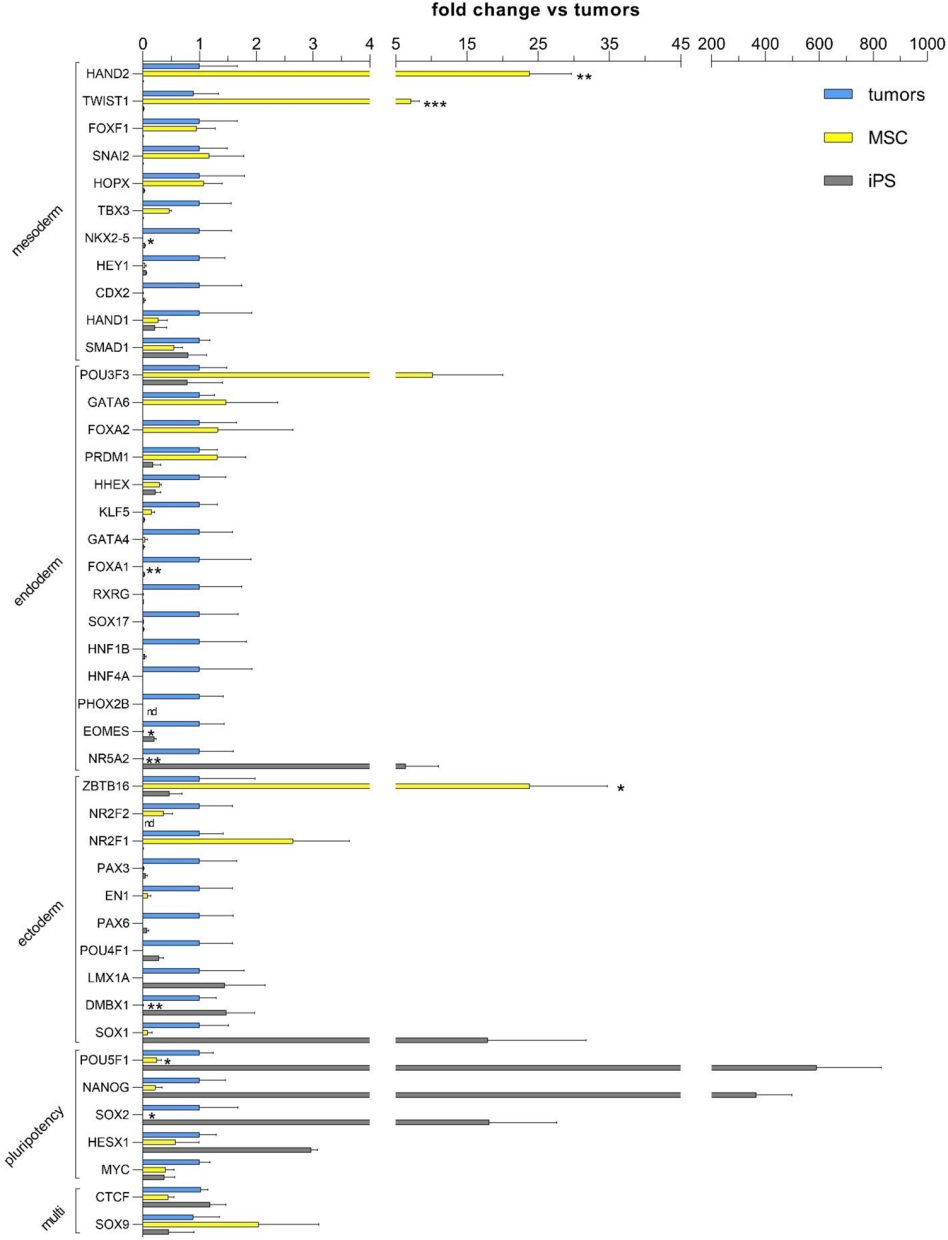
Expression of lineage-specific transcription factors and transcriptional regulators in all cancer cell lines, iPSCs, and MSC lines (*n* = 3 for cancer cell lines, *n* = 2 for iPSC cell lines, and *n* = 10 for MSC cell lines). 2-ΔΔCT values for each individual gene were averaged for biological groups and calculated as the fold change of cancer cells vs. MSCs and iPSC lines. Data are presented as the mean ± SEM. Statistical analysis was performed in GraphPad using the Mann–Whitney test; *p < 0.05, **p < 0.01, and ***p < 0.001; nd, not detected

**Fig. 3:**
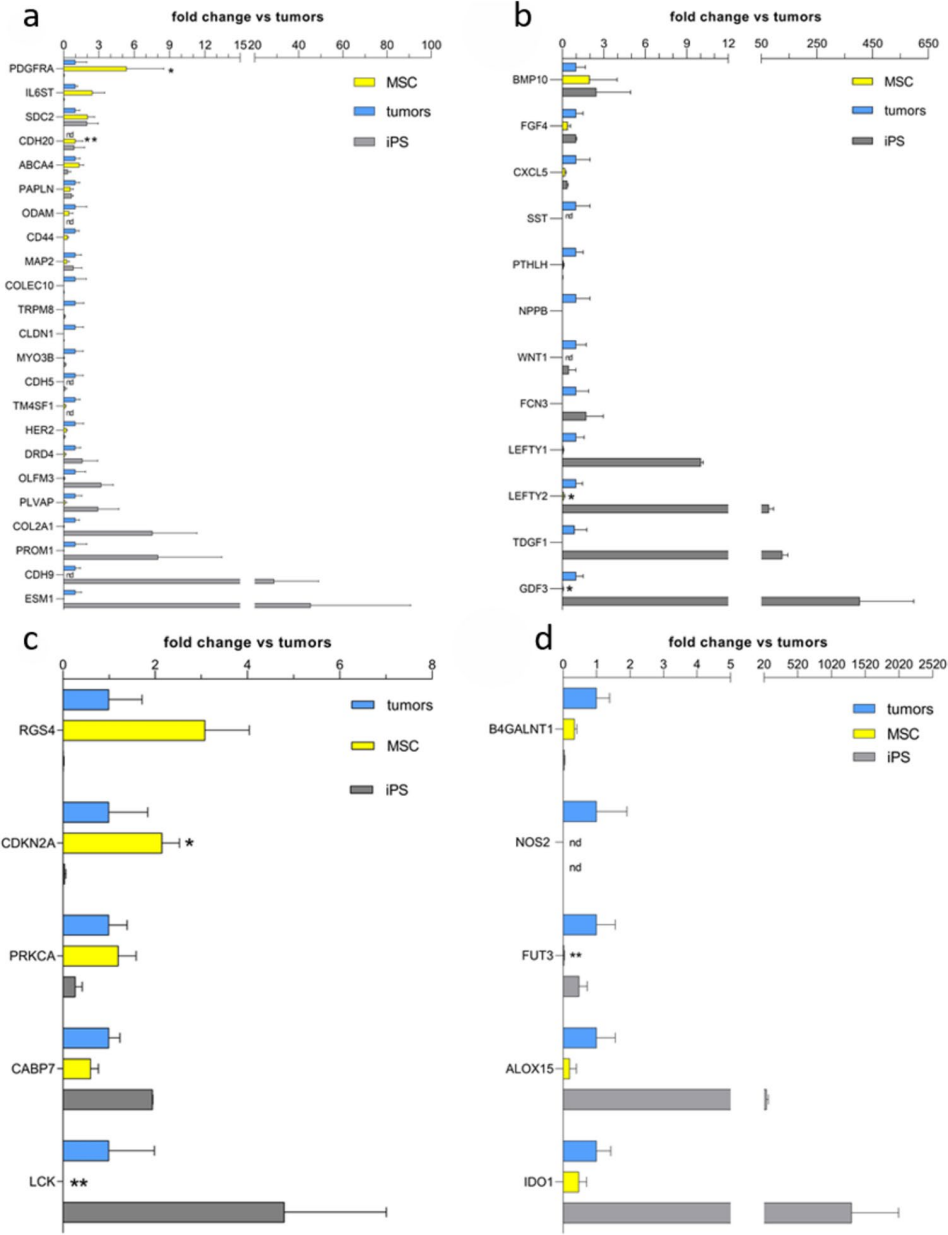
Expression of gene markers of different panels in cancer cell, iPSC, and MSC lines (*n* = 3 for cancer cell lines, *n* = 2 for iPSC cell lines, and *n* = 10 for MSC cell lines). The 2-ΔΔCT values for each individual gene were averaged for biological groups and calculated as the fold change in cancer cells vs. MSCs and iPSC lines, except for CDH20 in cancer cell lines for which all expression values were 0; thus, normalization was performed on iPS cell line expression. Gene coding membrane-associated and cell structure proteins are shown in **a**, **b** genes coding extracellular signaling proteins, **c** genes coding signal transduction proteins, and **d** genes coding metabolic regulator proteins. Data are presented as the mean ± SEM. Statistical analysis was performed using the Mann–Whitney test; *p < 0.05; **p < 0.01; nd, not detected

Additionally, the design included a group of 8 genes (MSC, pluripotency, and cancer cell markers) and two respective controls for data normalization, which were evaluated in a custom-designed assay using RT-PCR assay. The expression of a total of 100 genes was evaluated in three cultured MSC lines at passage three, two iPSC lines, and nine commercial cancer cell lines: ZR-75-30, A-375, A-431, HT-1080, MCF-7, MG-63, A-549, NCI -H727, and K1, of different embryonic origins (Fig. S1 supplementary materials). Based on normalized values of gene expression, we created gene panels according to biological origin and functions (Figs. 2 and 3). We compared the expression of 8 markers (MSCs, cancer cells, and pluripotent markers) in MSC lines from early (p3) and late passages (p10) in a custom-designed assay (Fig. 5).

The first biological panel (Fig. 2) consisted of gene coding for transcriptional regulators. The line-age-specific genes were panel-assigned based on the literature and the predefined Scorecard assignment (Bock et al. 2011). As predicted, MSC lines showed negligible or no expression of the core pluripotency transcription factors OCT4 (POU5F1), SOX2, or NANOG. Expression of HESX1 (homeobox expressed in ES cells 1), which encodes a transcription factor governing the pluripotent state (Li et al. 2015), was present in MSC lines, yet it was lower than that in the iPSC group. The expression of MYC, which encodes a transcription factor crucial for maintaining numerous signaling pathways and is a hallmark of active chromatin (Varlakhanova et al. 2010) in highly proliferative cells, was similar between MSCs, cancer cells, and iPSC lines.

OCT4 (POU5F1), SOX2, and NANOG were expressed at higher levels in cancer cell lines than in MSCs. This shows that MSCs do not express the essential transcription factors associated with self-renewal. Selected pluripotency genes underline differences between highly active iPSCs and less potent MSCs, yet with clearly marked proliferative capacity of the latter.

Three other marker sets consisted of genes coding for transcriptional regulators associated with ectoderm, endoderm, and mesoderm specification (Varlakhanova et al. 2010). The ectoderm panel of transcription factors showed that ZBTB16 (zinc finger and BTB domain-containing protein 16) was significantly overexpressed in MSCs compared to cancer cells and iPSC lines. In contrast, DMBX1 (Diencephalon/Mesencephalon Homeobox 1), which regulates cell cycle exit and differentiation of progenitor cells, was significantly downregulated in MSC lines. The endoderm panel of transcriptional regulators revealed that MSC lines did not express a large group of transcription factors, including GATA4, RXRG, SOX17, HNF1B, HNF4A, and PHOX2B, among which FOXA1 (forkhead box A1), EOMES (Eomesodermin), and NR5A2 (nuclear receptor subfamily 5 group A member 2) were significantly downregulated. The mesoderm panel of transcriptional regulators showed that MSC lines did not express CDX2 and HEY1 transcription factors. The expression of HAND2 (heart- and neural crest derivatives-expressed 2) and TWIST1 (twist family bHLH transcription factor 1) was significantly upregulated, while the expression of NKX2-5 (NK2 Homeobox 5) was significantly downregulated in MSCs compared to cancer cell lines. The expression of these markers was negligible in iPSC lines. The next large panel of markers consisted of genes coding for membrane-associated and cell structure proteins (Fig. 3a). This panel included PDGFR-α (platelet-derived growth factor receptor alpha) and CDH20 (cadherin 20); both were significantly upregulated in MSCs compared to cancer cell lines.

Markers consisting of genes encoding selected extracellular signaling proteins formed the second panel (Fig. 3b), allowing the comparison of the secretion activity between the groups. Except for BMP10 (bone morphogenetic protein 10), MSC lines did not express markers included in this panel, among which LEFTY2 (left-right determination factor 2) and GDF3 (growth differentiation factor-3) were significantly downregulated.

The typical pluripotency marker teratocarcinoma-derived growth factor 1 (TDGF1) was highly overexpressed in iPSC lines. Additionally, LEFTY1, LEFTY2, and GDF3 showed higher expression in iPSC lines than in cancer cells and MSC lines.

Genes coding for proteins involved in signal transduction, including kinases CDKN2A (cyclin-dependent kinase inhibitor 2A), PRKCα (protein kinase C-alpha), LCK (Leukocyte C-Terminal Src Kinase), signal transduction regulators RGS4 (regulator of G protein signaling 4), and CABP7 (calcium-binding protein 7), were grouped into the next panel and are presented in Fig. 3c. CDKN2A was significantly upregulated, while LCK, key gene in the maintenance of the undifferentiated state, was significantly downregulated in MSCs vs. cancer cells and iPSC lines.

Metabolic control is crucial for maintaining pluripotency and cancer cell growth. We created a panel of metabolic regulators (Fig. 3d). FUT3 (fucosyltransferase 3), which plays a role in the fucosylation of glycosphingolipids, was significantly downregulated in MSCs compared to cancer cell lines. In contrast, IDO1 (indoleamine 2,3-dioxygenase 1), which is involved in tryptophan metabolism, and ALOX15 (arachidonate 15-lipoxygenase), which regulates the metabolism of polyunsaturated fatty acids, were highly overexpressed in iPSCs compared to MSCs and cancer cell lines. B4GALNT1 (be-ta-1,4-N-acetyl-galactosaminyltransferase 1) showed low expression in both MSCs and iPSCs compared to cancer cell lines.

**Fig. 4:**
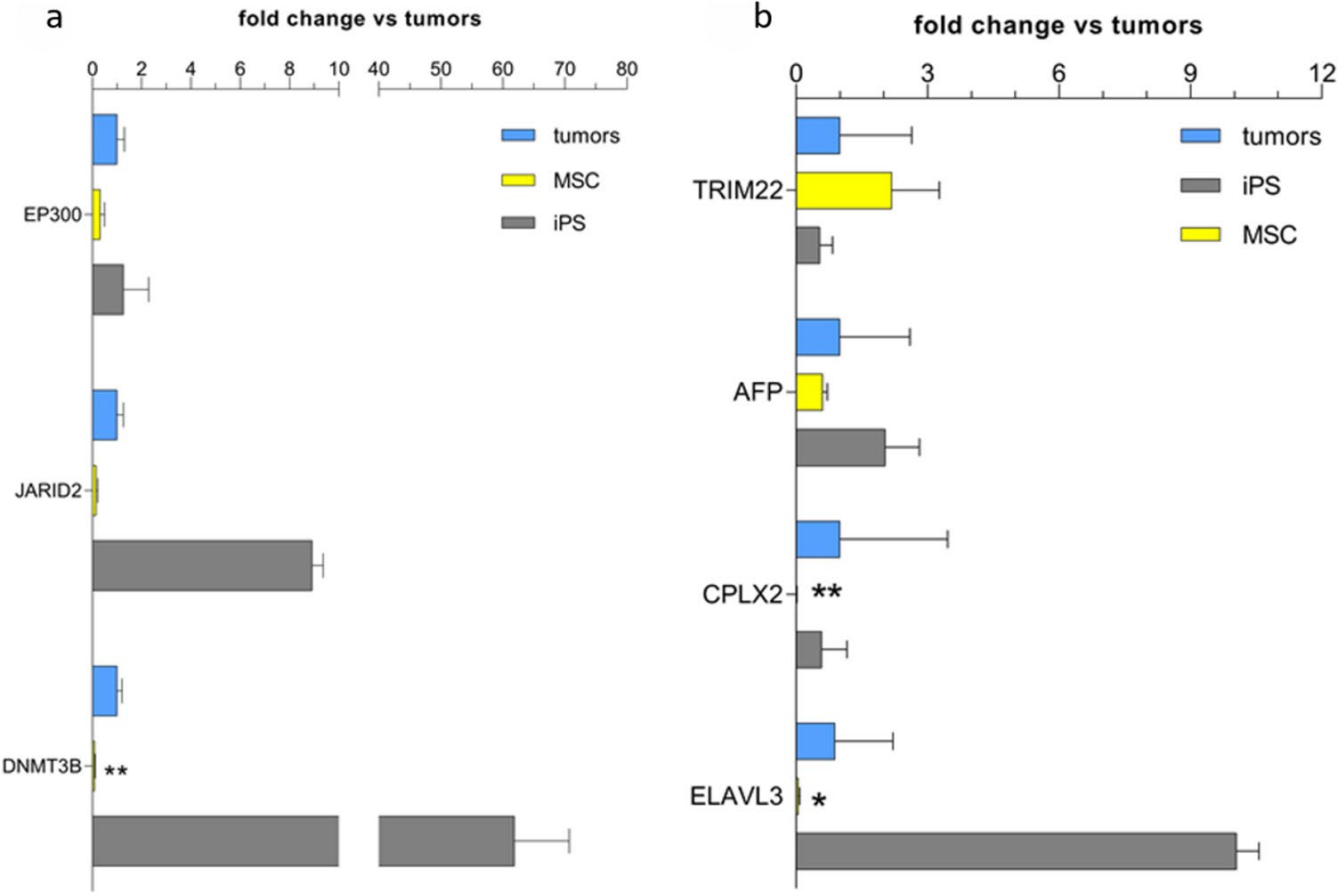
Expression of gene markers of different panels in cancer cells, iPSC, and MSC lines. The 2-ΔΔCT values for each individual gene were averaged for biological groups and calculated as the fold change in cancer cells vs. MSCs and iPSC lines, except for CDH20 in cancer cell lines for which all expression values were 0. Gene coding epigenetic regulator proteins (**a**) and other genes (**b**). Data are presented as the mean ± SEM. Statistical analysis was performed using the Mann–Whitney test; *p < 0.05; **p < 0.01; nd, not detected

Of the 3 genes included in the panel of epigenetic regulators, DNMT3B (DNA methyltransferase 3 beta) and JARID2 (Jumonji and AT-rich interaction domain containing 2) were overexpressed in iPSCs compared to MSCs and cancer cell lines (Fig. 4a). Additionally, only DNMT3B was significantly downregulated in MSCs vs. cancer cell lines. Levels of EP300 (histone acetyltransferase P300) did not differ between MSCs, cancer cells, and iPSC lines.

The last group of genes included TRIM22 (tripartite motif-containing 22), AFP (alpha-fetoprotein), CPLX2 (complexin-2), and ELAVL3 (ELAV-like RNA binding protein 3). CPLX2 and ELAVL3 were significantly downregulated in MSC lines compared to cancer cell lines (Fig. 4b). Interestingly, the expression of ELAVL3 was high in iPSC lines.

**Fig. 5:**
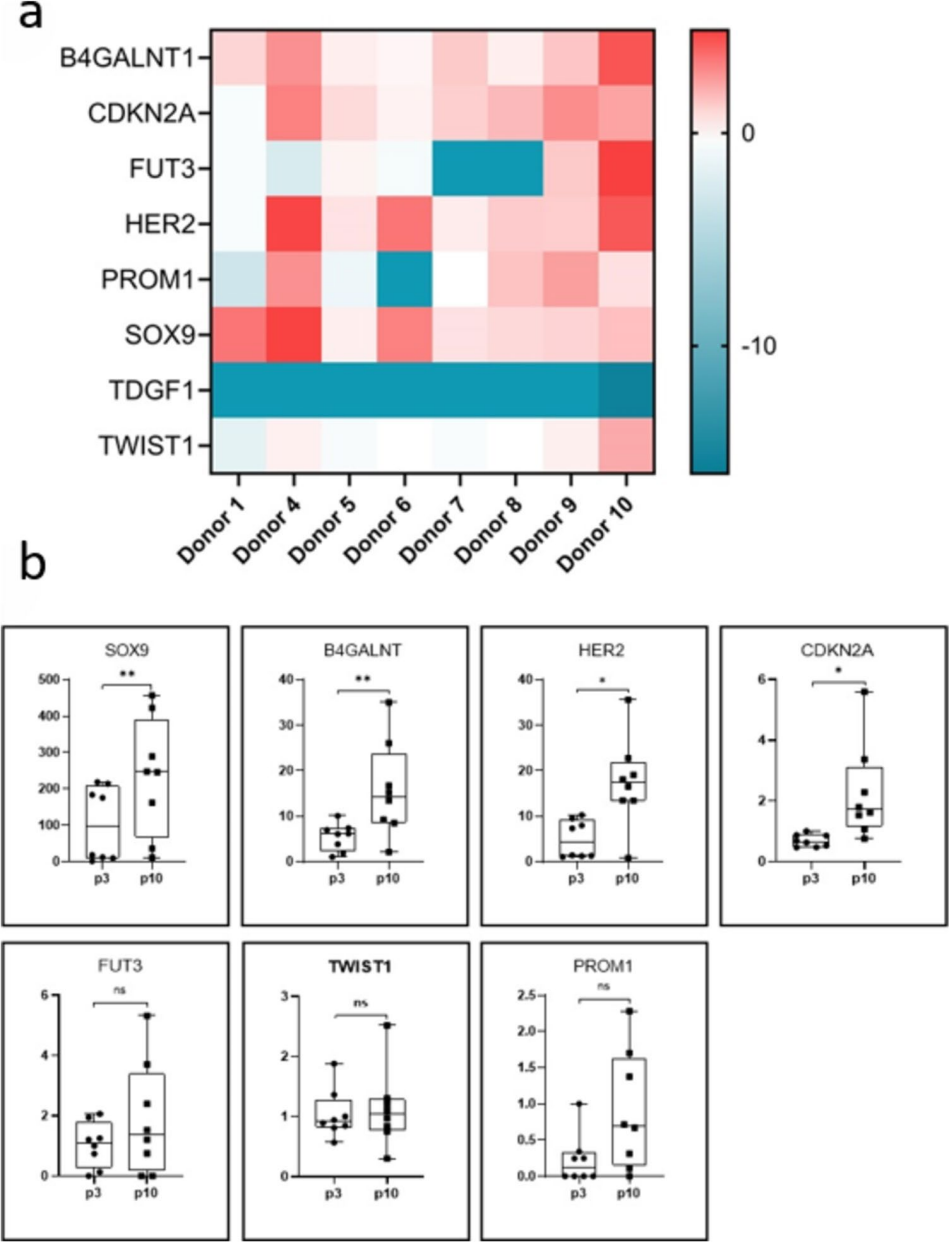
Comparison of marker relative expression in MSCs of early (p3) to late (p10) passages (8 independent lines) based on a custom-designed assay. **a** A comparison of relative gene expression from late passage (p10) to early passage (p3) for each donor. The 2-ΔΔCT values were log2 transformed to obtain a uniform distribution in the heatmap presentation. Due to the minimal expression of the TDGF1, it represents the lowest values on the heat map. Genes that were amplified beyond Ct 36 were considered negative and were assigned the lowest value, − 16,61. **b** Graphs showing the expression of pluripotency, MSCs, and 2-ΔΔCT values were prepared in GraphPad. Data are presented as box plots incorporating individual values with the mean ± SEM. Statistical analysis was performed in GraphPad using the Mann–Whitney test; *p < 0.05; **p < 0.01; ns, not significant

We performed an analysis of gene expression changes in early (p3) and late (p10) MSC passages mimicking the process of cell aging in *in vitro* culture while using an extended MSC population (*n* = 8). The process of MSC aging has been investigated in previous manuscripts (Sethe et al. 2006). The selected gene panel was created based on the literature and includes B4GALNT1, CDKN2A, FUT3, HER2, PROM1, SOX9, TDGF1, and TWIST1. Within this group, SOX9, TWIST1, and CDKN2A have been previously reported to be variable in aging cell populations or significantly affect cell proliferation and differentiation (Li et al. 2017, Cakouros et al. 2012). Our direct comparison revealed that the expression of B4GALNT1, CDKN2A, HER2, and SOX9 increased significantly as cells aged (Fig. 5).

## Discussion

MSCs were first applied in a clinical trial over a decade ago. Since then, MSCs have been used in several therapeutic approaches (Kobolak et al. 2016). There is a strong need for a universal strategy confirming the safety of MSCs, including evaluation of tumorigenicity risk, which has thus far been proven and linked mainly to pluripotent cell therapy products (ESCs and iPSCs) (Hentze et al. 2009, Yasuda et al. 2018). Although compared to pluripotent stem cells, MSCs have a significantly lower risk of oncogenic events, current methods to evaluate their safety still lack sensitivity. To address stem cell safety regulations, numerous organizations have been established, such as the Health and Environmental Sciences Institute (HESI) and the Committee for Nonclinical Safety Evaluation of Pluripotent Stem Cell-derived ProducT (FIRM-CoNCEPT) (Sato et al. 2019).

The main guidelines for culturing MSCs for therapeutic use were described by the ISCT. Additionally, two techniques were suggested to identify genetic errors: karyotyping and molecular arrays (Sato et al. 2019). Both are used to detect recurrent abnormalities but on different levels of sensitivity, which may cause more subtle mutations to be missed and thus increase the potential risk of tumorigenicity.

In this manuscript, we attempted to find novel ways to assess WJ-MSC tumorigenic potential. After reviewing the available literature, we selected 100 genes whose expression level was analyzed in WJ-MSC lines vs. cancer cells and iPSC lines based on a hPSC Scorecard gene panel and additional literature-based markers to define genes that help to distinguish these cell lines at the molecular level. Our WJ-MSC lines showed negligible expression of typical iPSC markers, such as TDGF1, LCK, DNMT3B, IDO1, OCT4 (POU5F1), SOX2, and NANOG. Previous studies detected the expression of ESC markers, such as SOX2, NANOG, TDGF1, and OCT4 (POU5F1) in MSCs; however, their relative expression was many times lower than that in ESCs (Riekstina et al. 2009, Heo et al. 2016, Fong et al. 2011, Gao et al. 2013). OCT4 (POU5F1), SOX2, and NANOG promote pluripotency and self-renewal through positive regulation of their genes and transcription factors (e.g., STAT3) or components of TGF-β signaling (e.g., TDGF1 and LEFTY2) (Boyer at al. 2005). Accordingly, we found that MSCs showed lower expression of LEFTY2 than iPSCs and cancer cells.

Metabolic programs are regulated by transcription factors, and in turn, metabolism controls the expression of fate regulators. ESCs and iPSCs rely mainly on glycolysis, which generates metabolites important for epigenome modifications. For example, acetyl-CoA produced by glycolysis maintains histone acetylation and pluripotency (Moussaieff et al. 2015). Overexpression of IDO1 in ES cells increases the levels of acetyl‐CoA and histone acetylation. IDO1 promotes glycolysis and pluripotency and is downregulated during the onset of hESC differentiation (Liu et al. 2019). We found that MSCs had reduced levels of IDO1 compared to iPSC and cancer cell line populations, although this was not significant. ESC renewal and differentiation are regulated by DNMT3B, a DNA methyltransferase. DNMT3B-null ESCs exhibit a disturbed mitochondrial fission and fusion balance and a switch from glycolysis to oxidative phosphorylation (Cieslar-Pobuda et al. 2020). Consistently, our MSC lines were devoid of DNMT3B expression. While iPSCs rely on glycolysis, in MSCs, there is a metabolic shift towards oxidative phosphorylation during expansion in culture in a nutrient-rich environment (Pattappa et al. 2011). Elevated glycolytic metabolism in ESCs is accompanied by increased MYC transcriptional activity and increased nuclear N-MYC and C-MYC levels (Gu et al. 2016). The MYC levels fluctuated only moderately between cancer cell, iPSC, and MSC lines. An additional aspect discriminating MSCs and iPSCs is proliferation capacity. While iPSCs maintain unlimited proliferation potential, MSCs following multiple passages acquire a senescent phenotype (Gu et al. 2006). The core transcription factors regulating pluripotency, NANOG, SOX2, and OCT4 (POU5F1), have been shown to regulate the expression of cell cycle-related genes, including positive and negative cell cycle regulators. These control pluripotency by multiple mechanisms, including feedback loops to SOX2 (Zaveri and Dhawan 2018). In our report, MSCs do not express pluripotency markers (or express them to a very limited extent); thus, MSCs cannot maintain the sustainable self-renewal stage.

We analyzed a group of genes that were upregulated in MSC lines compared to cancer cell lines, including transcription factors (ZBTB16, HAND2, and TWIST1), a cadherin regulating cell adhesion (CDH20), a receptor tyrosine kinase (PDGFR-α), and a negative cell cycle regulator (CDKN2A). These genes showed limited or no expression in iPSC lines. TWIST1 is a helix-loop-helix transcription factor. While the levels of TWIST1 were consistent among the MSC lines analyzed in this study, a previous report showed that donor-to-donor differences in TWIST1 levels correlated with MSC viability (Boregowda et al. 2015). In the same study, TWIST1 was shown to regulate gene networks controlling cell surface receptor-linked signal, transduction, cell division, and DNA replication. Intriguingly, TWIST1 regulates the expression of the p14/p16 locus in human bone marrow stromal stem cells (Cakouros et al. 2012). The authors presented a model in which high levels of TWIST1 during an early passage repress the Ink4A/Arf locus resulting from increased histone methylation by recruitment of histone methyltransferase, EZH2. As the cells senesce, the levels of TWIST1 decrease, resulting in transcriptional activation of p14/p16 (Cakouros et al. 2012). We found elevated expression of CDKN2A (coding for p14/p16) in MSCs compared to cancer cells and iPSC lines. While the levels of TWIST1 did not differ between early- and late-passage cells in our experiment, increased CDKN2A expression at later passages was observed. Transcription factors, HAND2, and its interaction with GATA4 and NKX2.5 are essential for cardiac morphogenesis. The interaction of HAND2 with TWIST1 and TWIST2 is important for limb development. HAND2 was expressed at higher levels in MSC lines than in iPSCs and cancer cell lines. HAND2 gene hypermethylation and epigenetic silencing increase the risk of endometrial cancer development (Jones et al. 2013). While we found HAND2 among genes upregulated in MSCs, HAND2 was previously not detected in human bone marrow-derived MSC lines (Riekstina et al. 2009). The difference may be attributed to the distinct tissue sources of MSCs. CDH20 (cadherin 20) has been shown to act as a cancer suppressor, and some cancer cell lines (e.g., melanoma cell line) lack the expression of CDH20 (Moore et al. 2004). CDH20 interacts with β-catenin, causing a reduction in the phosphorylation and nuclear translocation of Smad2/3, thus suppressing transforming growth factor-β (TGF-β) signaling (Li et al. 2020) and partially blocking the epithelial to mesenchymal transition (EMT) by downregulating the Snail/Smad2/3 pathway. We show that MSCs express CDH20, thus presenting another security switch for cell cycle control and repression of potential oncogenic transformation. Comparison of gene expression in the custom-designed assay showed that the expression of B4GALNT1, SOX9, and HER2 increased during later passages. B4GALNT1 encodes β−1,4-N-acetyl-galactosaminyltransferase 1, a key enzyme in generating gangliosides GM2/GD2, and plays a role in regulating cell adhesion (Martinez et al. 2007). The expression of B4GALNT1 in our MSC lines also increased during later passages. Previous studies reported that B4GALNT1 is expressed by MSCs derived from different tissues (Heo et al. 2016, Martinez et al. 2007). A study showed that the SOX9 transcription factor is expressed at similar levels in bone marrow-derived MSCs as in ESCs (Riekstina et al. 2009). Despite its role in chondrogenesis, it plays an important role in cell survival and proliferation of MSCs by controlling the levels of cell cycle regulators (Stöck et al. 2013). HER2 is a receptor tyrosine-protein kinase that regulates cell signaling, and it is a known cancer marker. It is also present in bone marrow MSCs, and its expression is enriched in a gene cluster related to proliferation (Khong et al. 2019).

Finally, we pinpointed significant overexpression of receptor tyrosine kinase alpha (PDGRF-α), a protein-coding gene responsible for mediating important cellular processes, such as proliferation, cell growth, and differentiation (Farahani and Xaymardan 2015), by activation of multiple downstream pathways, including VEGF, PI3K/Akt, and PLCγ/PKC. Some reports state that abundant amounts of PDGFR-α are a characteristic feature of undifferentiated MSCs, in which it acts as a differentiation inhibiting factor and plays critical functions during embryonic development (Ball et al. 2007). Thanks to the important crosstalk between VEGF-A and PDGFR-α, PDGFR-α is responsible for MSC migration and plays a role in neovascularization (Farooqi and Siddik 2015). Although it may be implicated during tumorigenesis due to the promotion of vasculogenesis, overexpression of PDGRF-α in mature tissues is now suggested as a novel marker of tumors. Based on Farooqi et al. (2015), PDGFR-α is repressed by p53 to regulate both neovascularization and cellular proliferation.

Using String software, we analyzed interaction networks for all panels of protein-coding markers. In this approach, the String analysis was inevitably biased, due to the use of a randomized but preselected pool of genes with overrepresented groups of genes concerning cell development and differentiation. Nevertheless, the analysis showed that panels shared a common regulator, p53. The tumor suppressor p53 regulates cellular processes, such as cell cycle control, differentiation, and DNA repair. Mutations in the p53 gene lead to genome instability and alterations in cell proliferation, the most common cancer-related genetic defect. MSCs are sensitive to mutations in the p53 gene and negative cell cycle regulator p21 (CDKN1A) (Farahani and Xaymardan 2015).

Loss of p53 expression in the absence of p21 expression in MSCs resulted in the formation of fibrosarcomas (Farahani and Xaymardan 2015), and it might have a connection with PDGFR-α-dependent tumorigenesis. Accordingly, it was shown that normal p53 expression is indispensable for MSC integrity (Boregowda et al. 2018). CDKN2A, coding for p16INK4A and p14ARF, acts as a negative regulator of the proliferation of normal cells. p16INK4A interacts with cyclin-dependent kinases 4 and 6 (CDK4 and CDK6). p14ARF blocks the activity of MDM2, an E3 ubiquitin ligase that regulates the activity and stability of p53. Because p53 is important for iPSC self-renewal (Abdelalim and Tooyama 2012), elevated expression of CDKN2A may be a marker that distinguishes MSCs from iPSCs and cancer cell lines. Accordingly, CDKN2A expression increased during later passages of MSCs, again suggesting that MSCs do not maintain the ability to proliferate indefinitely. Thus, the elevated expression of negative cell cycle regulators such as CDKN2A may be a key determinant of the limited proliferation potency of MSC lines.

In this study, researchers compared 98 genes for their expression in MSC together with cancer lines of different embryonic origin and IPS lines. The goal was to find genes that distinguish MSCs when compared to other cell populations (cancer cell lines and iPSCs) which would allow substantial improvements to currently used methods of identifying the population of MSC cells. On the other hand, detection of characteristic markers distinguishing both pluripotent and cancer cells would significantly improve the safety of preparations based on MSC due to their negative selection. In this study, researchers managed to detect over a dozen such genes, which may be the basis for the further development of the identification panels. Among the examined genes, MSCs showed the greatest difference in expression compared to other lines in such genes as PDGRF-a, CDH20, and CDKN2A. IPS lines were characterized by significant overexpression in the case of genes generally considered as pluripotency markers such as DNMT3B, IDO1, LCK, and TDGF1 but also GDF3 or ELAVL3. In addition, although it was not in the spectrum of main interest, a set of potentially usable genes for distinguishing characteristically MSC lines from cancer lines was detected, including those of different embryonic origins such as FUT3, NOS2, COLEC10, TRPM8, CLDN1, MYO3B, CDH5, TM4SF1, SST, PTHLH, and NPPB. However, the type of analysis presented here leads to the loss of information about the specific differences between MSC and cancer cell line expression. Selected genes, when averaged, show the most prominent tumorigenicity vs. MSC markers that are stable regardless of the tumor type. Due to the differences, these genes are most probably related to the mechanisms of uncontrolled proliferation, which is a key feature of tumors. Thus, there is a high chance that the cancer cell markers selected based on presented approach will be highlighted similarly in any other randomly selected cancer.

We proposed the use of selected genes to create a gene panel to characterize MSC populations and exclude their overlapping with cancer cell line expression. This could further increase confidence in patients, physicians, and manufacturers, concerning the quality and safety of products based on MSC. Diversity of MSC gene expression profile depends on the culture medium and isolation site. In this study, we aimed to ensure that cells did not exhibit gene expression similar to cancers, with specific for MSC gene expression within the range.

A fine-tuning of the designed tool requires more samples analyzed also from other than umbilical blood cord source of MSC, to create a fully reliable assay.

## Conclusions

In summary, we characterized the expression profile of different WJ-derived MSCs and compared them to select cancer cell lines and iPSCs. We isolated various genes that differentiate WJ-MSCs from cancer cells and iPSC lines. Moreover, we identified distinct transcriptional, metabolic, and cell cycle regulators of MSCs, cancer cells, and iPSCs.

Finally, the genes described in the manuscript can provide a strong basis for the development of a panel that could be used as a molecular test to define the origin and tumorigenicity of WJ-MSCs in clinical applications.

### Patents

Based on the data presented in this article, the patent has been filed with the European Patent Office.

## Supplementary Information

Below is the link to the electronic supplementary material.

Supplementary file1 (DOCX 284 KB)

## Data Availability

The data presented in this study are available on request from the corresponding author.
