# Supplementary material for: Development of a novel gene expression panel for the characterization of MSCs for increased biological safety
Source: J Appl Genet. 2024 Dec 2;66(3):623–36. doi: 10.1007/s13353-024-00917-5 (PMC12367896; doi:10.1007/s13353-024-00917-5)
Supplement: Supplementary file 1 — Supplementary file1 (DOCX 284 KB) [file 13353_2024_917_MOESM1_ESM.docx]

**Supplementary materials**


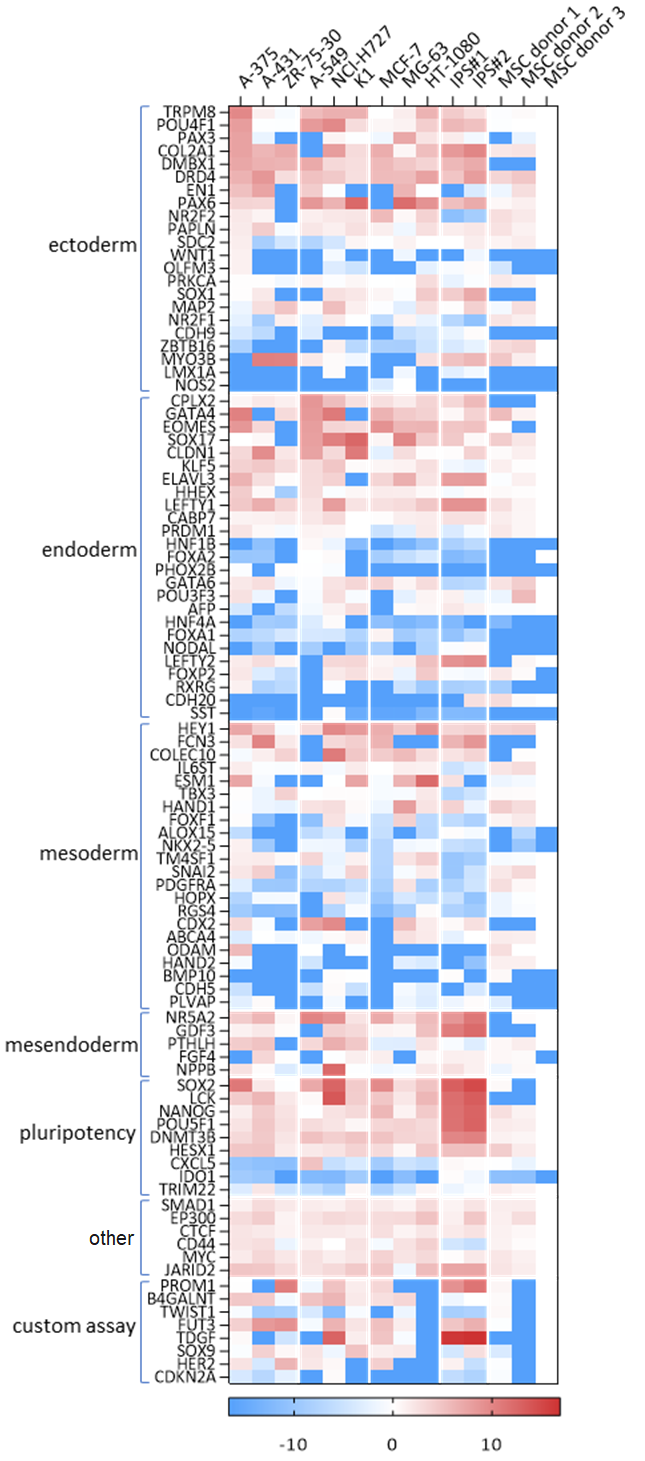


**Figure S1**. Heat map of gene marker expression based on custom designed assays in cancer cell line, iPSCs and MSCs. 2^-ΔΔCT^ values were log2 transformed to obtain uniform distribution in the heatmap presentation.
Genes which amplified beyond Ct 36 were assigned as negatives with the lowest value -16,61. MSCs at passage 3
(3 independent donors) vs iPSCs (2 independent lines) vs cancer cell lines ZR-75-30, A-375, A-431, HT-1080, MCF-7, MG-63, A-549, NCI -H727, K1. Genes were grouped according to predefined hPSC Scorecard™.

**Table S1**: Sequences of designed primers for qPCR analyses.

| **Gene** | **Direction** | **Sequence** |
| --- | --- | --- |
| **PROM1** | Forward | TACAACGCCAAACCACGAC |
|  | Reverse | TGAATAGGAAGACGCTGAGTTACA |
| **CDKN2A** | Forward | GCACTCACGCCCTAAGC |
|  | Reverse | TCAAGAGAAGCCAGTAACCCC |
| **FUT3** | Forward | GTCCCGCTGTTCAGAGATGG |
|  | Reverse | GAGGCGTGACTTAGGGTTGG |
| **TDGF1** | Forward | CCTTCTACGGACGGAACTGT |
|  | Reverse | GAAGCCACGAGGTGCTCATC |
| **HER2** | Forward | GGGAAACCTGGAACTCACCT |
|  | Reverse | GGGACCTGCCTCACTTGG |
| **SOX9** | Forward | GGACCAGTACCCGCACTTG |
|  | Reverse | GTTCTTCACCGACTTCCTCCG |
| **B4GALNT1** | Forward | CCCTCAGGAGCATCTTGGTG |
|  | Reverse | TAGGGAGGCAGTCAGGTTCA |
| **TWIST1** | Forward | CTCAGCTACGCCTTCTCGGT |
|  | Reverse | TCCTTCTCTGGAAACAATGACATCT |
| **Reference genes** |  |  |
| **GAPDH** | Forward | TGAAGGTCGGAGTCAACGG |
|  | Reverse | CTGGAAGATGGTGATGGGATTT |
| **HPRT1** | Forward | CTGGCGTCGTGATTAGTGATGA |
|  | Reverse | GAGGGCTACAATGTGATGGC |
